# Obstacles à la conduite d'une enquête d'imputabilité d'une toxidermie médicamenteuse selon la méthode de Bégaud en psychiatrie. A propos d'un cas clinique et d'une revue de la littérature

**DOI:** 10.11604/pamj.2014.17.222.2272

**Published:** 2014-03-21

**Authors:** Djibo Douma Maiga, Laouali Salissou, Mamane Daou

**Affiliations:** 1 Faculté des sciences de la santé de l'université Abdou Moumouni de Niamey. Département de Médecine et spécialités médicales de l'hôpital National de Niamey; 2 Département de Médecine et spécialités médicales de l'hôpital National de Niamey

**Keywords:** Toxidermie, psychotropes, enquête d'imputabilité, méthode de Bégaud, Niamey, Toxiderma, psychotropic, imputability investigation, Bégaud method, Niamey

## Abstract

Nous rapportons un cas de toxidermie médicamenteuse dans un contexte de poly médication de psychotropes. La difficulté de l'enquête d'imputabilité de la ou des substances en cause(s) selon la méthode de Bégaud est mise en exergue dans notre contexte. Cette difficulté est liée à la représentation de la maladie mentale d'une part, et à la faible fréquence des accidents dermatologiques liée aux substances en causes d'autre part. Il apparaît que seule une prescription judicieuse de médicaments psychotropes en psychiatrie peut amoindrir le risque d'accidents dermatologiques et son corollaire d'enquête d'imputabilité à résultat aléatoire.

## Introduction

Les toxidermies sont les complications cutanéo-muqueuses secondaires à l'administration par voie entérale, intraveineuse, sous-cutanée ou intramusculaire de médicaments [1]. Certains médicaments psychotropes utilisés en psychiatrie, sont reconnus responsables de réactions secondaires dermatologiques parfois graves [2–9]. Si le diagnostic clinique de la réaction dermatologique est aisé, cependant la conduite de l'enquête de l'imputabilité du ou des médicament (s) absorbé (s) par le patient peut être difficile en raison de la prescription fréquente de plusieurs psychotropes.

Nous rapportons un cas de toxidermie médicamenteuse chez une patiente ayant reçu Phénobarbital, Halopéridol, Chlorpromazine, Trihexiphénidyle, Ampicilline et Diazépam. L'objet de ce rapport, qui s'appui également sur une revue de la littérature, est de souligner la difficulté de la conduite de l'enquête d'imputabilité selon la méthode de Bégaud [9] et l'intérêt préventif d'une prescription judicieuse de médicaments psychotropes dans notre contexte.

## Patient et observation

Mademoiselle A.Z. 23 ans a été vue en consultation le 09/09/2011 pour crises convulsives évoluant depuis plusieurs mois. Ces crises répondaient aux critères diagnostics d'épilepsie. Elle a reçu une prescription de 200mg de Phénobarbital à prendre en une prise vespérale. Elle a été revue le 07/10/2011à la demande de ses parents, et hospitalisée pour des symptômes d'allure psychiatriques (instabilité psychomotrice, agressivité physique importante, logorrhée, obnubilation et désorientation temporospatiale). Ces symptômes n'ont pas été contrôlés par l'adjonction de Diazépam (60 mg par jour). Un traitement antipsychotique (Halopéridol 20 mg et Chlorpromazine 400 mg en deux prises par jour) a été mis en place. Un antiparkinsonien (Trihexiphénidyle) a été prescrit pour prévenir les effets secondaires neurologiques des neuroleptiques. Elle avait quitté l'hôpital le 14/10/2010 avec un état de santé cliniquement amélioré.

**Figure 1:**
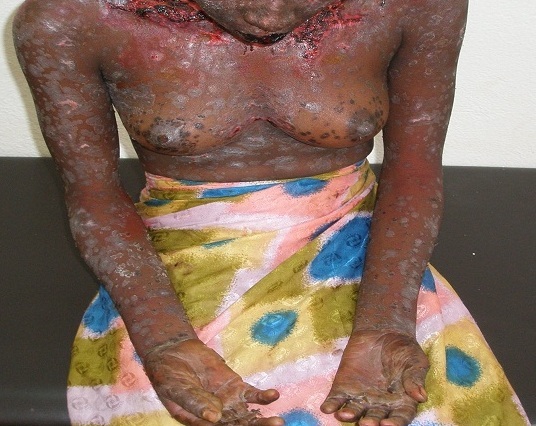
Fin du décollement bulleux et début d'assèchement des lésions

**Figure 2:**
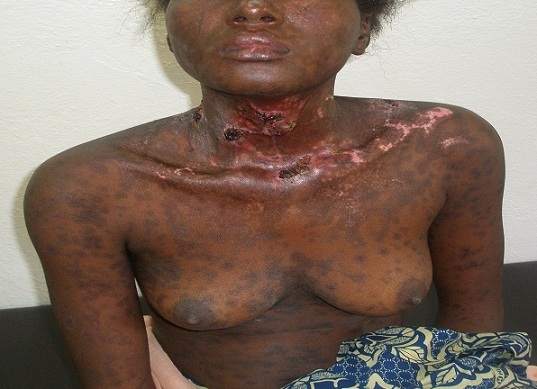
Chute des croutes et cicatrices tégumentaires érythémateuses

Une semaine après sa sortie, la patiente a été ramenée en urgence le 17/10/2011, dans un tableau de confusion mentale aigue. L'examen clinique chez cette patiente déshydratée, notait une fièvre à 40.5°c. Les muqueuses buccales et nasales étaient érythémato-pustuleuses et celles oculaires très érythémateuses avec larmoiement. L'examen biologique n'a montré qu'une anémie microcytaire hypochrome avec leucopénie. La conduite immédiate a été l'arrêt de tous les traitements antérieurs, la prescription d'une réhydratation (3litres par jour : quels solutés ') et un traitement antibiotique (3g d'Ampicilline) per os pour hypothèses diagnostiques de syndrome infectieux et d'hyperthermie maligne des neuroleptiques. Au 2^e^ jour de l'hospitalisation, le diagnostic de toxidermie médicamenteuse type nécrolyse épidermique toxique ou syndrome de Lyell a été évoqué devant l'apparition de décollements bulleux sur l'ensemble du corps (Figure 1). L'ampicilline a été remplacée par l'Erythromycine (2g par jour) et des soins locaux étaient institués (antiseptique nasale, auriculaire et cutané) en plus de la réhydratation. Au 5^e^ jour du traitement, l'évolution était favorable avec la diminution progressive du suintement cutané, la formation puis la chute des croutes, laissant parfois un tégument érythémateux analogue à celui d'une brulure de 2^e^ degré (Figure 2). Après 15 jours d'hospitalisation la patiente avait quitté l'hôpital avec une prescription de Valproate de sodium 2g en deux prises. Revue 2 semaines après sa sortie, l'examen clinique notait une irritabilité et une hypoacousie.

## Discussion

L'apparition des lésions érythémateuses ou érytémato-pustuleuses et des décollements bulleux sur l'ensemble du corps de notre patiente a fait suspecter le diagnostic de la toxidermie médicamenteuse type nécrolyse épidermique toxique ou syndrome de Lyell. Le premier obstacle n'était pas tant la mise en œuvre du deuxième volet, celui de la recherche de l'imputabilité intrinsèque selon la méthode de Bégaud [10], puisse que l'arrêt des médicaments en cours avait induit une évolution favorable des lésions cutanées, mais plus, celle du premier volet. En effet le biais d'écoute et d'observation du patient par le personnel médical en raison de sa stigmatisation, l'insuffisance des informations anamnestiques recueillies auprès de la patiente et de ses accompagnants n'avaient pas permit.

L'établissement du tableau chronologique de l'apparition de la symptomatologie. Il s'agit là de raisons, selon certains auteurs qui font obstacles l'évaluation somatique des personnes mentalement malades [11].

Relativement au tableau chronologique [1], on a pu seulement rejeter l'hypothèse d'une réaction allergique de type immédiate à l'un des cinq médicaments (Phénobarbital, Chlorpromazine, Halopéridol, Trihexiphénidyle, Diazépam) en raison du délai relativement long entre leur introduction dans le traitement et la survenue de l'événement. L'hypothèse de la responsabilité de l'Ampicilline avait également été rejetée car introduite après le début de l'événement. Il a semblé alors s'agir d'une réaction de type retardé immunologique ou non à l'une des cinq molécules (Phénobarbital, Chlorpromazine, Halopéridol, Trihexiphénidyle, Diazépam); ce qui avait justifié l'arrêt de tous les traitements. Le deuxième obstacle a été de déterminer la molécule responsable, puisse que prise singulièrement chacune peut être responsable de réactions cutanées et muqueuses [1, 4, 5, 10]. Cet obstacle n'a pas pu être levé sans l'apport discriminant de la biopsie et des tests cutanés qui ne constituent pas des pratiques médicales de routine à Niamey. Par ailleurs, le troisième obstacle représenté par la responsabilité d'éventuels facteurs infectieux endémiques dans notre contexte et de facteurs de prédisposition génétique ne pouvaient jamais être écarté. Il est resté à discuter l'imputabilité extrinsèque des ces molécules. Des cas de réaction dermatologique retardée allergique ou toxique ont été rapportés après ingestion de Phénobarbital [2]. Un érythème noueux polymorphe a été décrit avec la Trihexiphénidyle [4]. Une nécrolyse épidermique toxique due à la formation de sous-produit cytotoxique après une exposition à la lumière ultraviolette a été rapporté après ingestion de Chlorpromazine [5]. L'Halopéridol associé à la Carbamazépine a été également incriminé [10]. L'étude de la fréquence de survenue des accidents dermatologiques liés à ces molécules n'a pas permit de discriminer leur responsabilité. Au total l'enquête d'imputabilité (intrinsèque et extrinsèque) ne pouvait plaider qu'en faveur de la responsabilité de l'association de ces cinq molécules chez notre patient.

## Conclusion

La poly prescription a été responsable d'une iatrogénie médicamenteuse. Le cas clinique rappelle que, seule une prescription judicieuse de médicaments psychotropes en psychiatrie peut amoindrir le risque d'accidents dermatologiques et son corollaire d'enquête d'imputabilité à résultat aléatoire.
